# Pediatric versus adult cases of primary osseous sarcomas of the spine: A population-based analysis

**DOI:** 10.1016/j.xnsj.2026.100865

**Published:** 2026-02-14

**Authors:** Christopher J. Carron, Ali Ebada, Nicholas Bever, Mary Ashley Liu, Salah G. Aoun

**Affiliations:** Department of Neurological Surgery, University of Texas Southwestern Medical Center, Dallas, TX, United States

**Keywords:** Sarcomas, Spine, Vertebral column, Osteosarcoma, Ewing’s sarcoma, Chondrosarcoma

## Abstract

**Background:**

Primary osseous sarcomas of the spine provide a challenge due to their anatomic location and aggressiveness. While chemotherapy, radiation, and surgery are frequently employed as treatments for these neoplasms, their efficacy has not been compared between children and adults.

**Methods:**

Using the Surveillance, Epidemiology, and End Results database (2000–2021), 455 patients were identified with confirmed osteosarcoma, Ewing’s sarcoma, or chondrosarcoma. Demographic, tumor, and treatment characteristics were analyzed. Cox proportional hazard models were used to evaluate mortality predictors, and Kaplan–Meier survival analysis was performed.

**Results:**

For the entire cohort, increasing age (hazard ratio [HR]=1.03; 95% confidence interval [CI]=1.01–1.04; p<.001), an osteosarcoma diagnosis (HR=2.06; CI=1.17–3.61; p=.0019), and increasing tumor size (HR=1.01, CI=1–1.02, p=.007) increased mortality risk. For adults, age, an osteosarcoma diagnosis, male sex (HR=1.94; CI=1.07–3.52; p=.0297) and Black race (HR=3.16; CI=1.07–9.34; p=.0374) conferred a poor prognosis. Subtotal tumor resection was protective for adults (HR=0.29; CI=0.09–0.92; p=.0355). For children, only increasing age (HR=1.13; CI=1.03–1.23; p=.00678) decreased survival. Kaplan–Meier analysis revealed cohort-wide median cancer-specific survival (CSS) of 82 months, with 5- and 10-year survival rates of 53% and 47%. Adult median CSS was 34 months, with 5- and 10-year survival rates of 44% and 37%. Median CSS was not reached in the pediatric cohort, with 5- and 10-year CSS rates of 69% and 64%.

**Conclusions:**

Children experienced improved CSS compared to adults. While resections were associated with survival in adults, survival in pediatric patients was not significantly influenced by treatment-related variables. These findings suggest that patient age and histology should guide prognosis and treatment strategy.

## Introduction

Primary osseous sarcomas of the spine are a group of rare, aggressive malignancies, including, but not limited to, Ewing’s sarcoma, osteosarcoma, and chondrosarcoma, that account for less than 5% of all primary bone tumors [1]. Due to their anatomical location, these tumors present distinct clinical challenges such as nonspecific symptomatology, surgical complexity, variable response to chemoradiotherapy, and limited therapeutic window [[2], [3], [4]].

Prior literature has found that age at diagnosis is a prognostic indicator for these malignancies; pediatric patients display unique tumor biology, treatment responses, and overall survival (OS) outcomes compared to adults [[5], [6], [7], [8], [9]]. For example, prior studies of primary osseous sarcomas across multiple anatomical locations have found that pediatric patients with Ewing’s sarcoma often benefit from earlier detection and more aggressive systemic therapy, whereas adults more frequently present with advanced disease and inferior survival outcomes [5,10]. Similarly, in osteosarcoma, younger patients tend to demonstrate improved OS following en bloc resection and adjuvant chemotherapy compared to adults [11]. In contrast, pediatric cases of chondrosarcoma, although rare, are more often associated with higher-grade tumors and worse outcomes than those observed in the adult population [12]. However, given that prior studies often combine spinal and nonspinal cases, these observed differences have not been well-characterized specifically in the context of spinal tumors, where survival outcomes may differ substantially across age groups.

To date, no large-scale population-based analysis has compared the clinical features, treatment patterns, and survival outcomes between adult and pediatric patients with primary osseous sarcomas of the spine. The present study used the Surveillance, Epidemiology, and End Results (SEER) database to describe demographic, tumor, and treatment characteristics, as well as OS, among pediatric and adult patients with primary spinal Ewing’s sarcoma, osteosarcoma, or chondrosarcoma. Our aim is to explore potential age-related differences that may provide additional context for clinical decision-making in this rare patient population.

## Methods

### Study population

Data for this analysis were obtained from the SEER registry (2000–2021) of the National Cancer Institute. This registry contains data from 17 geographic areas in the United States, encompassing 26% of the US population [13]. Sarcoma cases in particular were identified through site and histology codes of the *International Classification of Disease for Oncology* (ICD-O-3). Diagnosis codes included in the analysis were as follows: 9,185/3: Small cell osteosarcoma; 9,260/3: Ewing sarcoma; 9,180/3; Osteosarcoma, NOS, 9,181/3; Chondroblastic osteosarcoma, 9,194/3; High grade surface osteosarcoma, 9,182/3; Fibroblastic osteosarcoma, 9,183/3; Telangiectatic osteosarcoma; 9,186/3: Central osteosarcoma; 9,184/3: Osteosarcoma in Paget disease of bone; 9,242/3: Clear cell chondrosarcoma; 9,240/3: Mesenchymal chondrosarcoma; 9,231/3: Myxoid chondrosarcoma; 9,243/3: Dedifferentiated chondrosarcoma; 9,221/3: Juxtacortical chondrosarcoma. Of these diagnosis codes, only patients with a confirmed location within the vertebral column (C41.2) were included.

Patient demographics, including age, sex, race, ethnicity, household median income, and marital status, were extracted. Disease characteristics such as tumor grade, tumor size, and histology were also included. Additionally, treatment characteristics such as receipt of radiotherapy, chemotherapy, surgery type (partial resection, total resection, no surgery), radiation/surgery sequence, time from diagnosis to treatment start, and year of diagnosis were also included. Outcome variables measured were survival in months, vital status, and year of follow-up. As SEER data is publicly available, this study was considered IRB exempt at our institution.

### Surgical procedure data

Surgery code review was carried out in accordance with the *SEER Program Code Manuals* [14]. In the SEER database, surgeries are coded according to previously established guidelines through review of medical records to establish the extent of tumor resection [15,16]. The variable “Rx Summ-Surg Prim Site (1998+)” contained these surgery codes for all included cases, which were then recoded manually into the following categories: (1) No surgery (code 00); (2) partial resection (codes 20, 25); (3) radical resection (codes 26, 40, 41); and (4) unknown (codes 19, 90).

### Statistical analysis

Statistical analysis was conducted entirely in *R version 4.2.3*. Continuous variables were reported as means with standard deviations, while categorical variables were summarized using counts and percentages. Survival analyses were conducted using the Kaplan–Meier method to estimate OS and cancer-specific survival (CSS), with differences between groups assessed using the exact log-rank test. Pediatric chondrosarcoma patients were excluded from stratified survivorship analysis due to insufficient sample size (*N*=5). CSS was defined as the time in months from diagnosis to death attributable to the cancer diagnosis, while OS was defined as the time in months from diagnosis to death attributable to any cause. Given the heterogeneity in patient age and comorbidities when comparing across adult and pediatric cohorts, CSS was selected as the primary outcome variable for regression analyses to better isolate the effects of tumor- and treatment-specific prognostic factors, minimizing confounding from unrelated mortality. Univariable and Multivariable Cox proportional hazard models (CPHM) were employed to understand the association between covariates and OS. Variables included in the analyses were: age at diagnosis (continuous, in years), sex (reference: female), race (reference: white), Hispanic ethnicity (reference: non-Hispanic), income quartile (reference: Q1: <$55K), histologic subtype (reference: Ewing sarcoma), tumor grade (reference: Grade II), surgical treatment type (reference: no surgery), radiation modality (reference: no radiation), radiation timing sequence (reference: no radiation after surgery), chemotherapy receipt (reference: no chemotherapy), and time to treatment (continuous, in months). All races aside from Black and white were combined into one subgroup entitled “Asian/Pacific Islander/American Indian” to assure adequate sample size. Hazard ratios (HRs) and corresponding 95% confidence intervals (CIs) were reported. All statistical tests were two-sided, and a p value <.05 was considered statistically significant.

For the pediatric subset, slight modifications were made to the analyses. Specifically, tumor size was removed as a covariate for the CPHM in order to preserve model stability due to a large number of missing values. For tumor grade, Grade I was grouped with “Unknown” in order to ensure sufficient sample size in each group. Of the 165 patient subset, there were 0 pediatric patients with Grade II tumors; thus, that group was removed from the analysis, and the reference group was changed to Grade III tumors.

## Results

### Patient demographics and treatment characteristics

455 patients with a diagnosis of osteosarcoma, Ewing’s sarcoma, or chondrosarcoma of the vertebral column and spinal cord were identified. These patients were then divided into adult (>18 years old, *N*=290) and pediatric (≤18 years old, *N*=165) subsets. The adult group had a mean age of 48 years (range 22–90 years, SD 18.6 years) while the pediatric group had a mean age of 12 years (range 0–17 years, SD 4.4 years). The majority of the cohort was white (80.9%) and male (61.3%); adults more commonly had osteosarcoma (47.6%), while children more commonly presented with Ewing’s sarcoma (83.6%). Surgical interventions included no surgery (30.3%), partial resection (42.4%), and radical resection (19.1%). Race, sex, ethnicity, median household income, and time from diagnosis to treatment were not associated with mortality in any other statistical analysis. A detailed representation of all demographic and treatment characteristics can be found in Table 1.

**Table 1:** Demographic and clinical characteristics of the entire cohort, pediatric patients, and adult patients.

Variable	Level	Whole cohort *N*=455 (%)	Adult *N*=290 (%)	Pediatric *N*=165 (%)	p value
**Sex**	Female	176 (38.7)	106 (36.6)	70 (42.4)	.256
	Male	279 (61.3)	184 (63.4)	95 (57.6)	
**Diagnosis**	Chondrosarcoma	43 (9.5)	38 (13.1)	5 (3)	<.001
	Ewing sarcoma	251 (55.2)	114 (39.3)	137 (83)	
	Osteosarcoma	161 (35.4)	138 (47.6)	23 (13.9)	
**Race**	American Indian/Alaska Native	6 (1.3)	3 (1.0)	3 (1.8)	.548
	Asian or Pacific Islander	35 (7.7)	26 (9.0)	9 (5.5)	
	Black	39 (8.6)	27 (9.3)	12 (7.3)	
	Unknown	7 (1.5)	4 (1.4)	3 (1.8)	
	White	368 (80.9)	230 (79.3)	138 (83.6)	
**Grade**	I	9 (2.0)	7 (2.4)	2 (1.2)	.070
	II	14 (3.1)	14 (4.8)	0 (0)	
	III	40 (8.8)	27 (9.3)	13 (7.9)	
	IV	78 (17.4)	51 (17.6)	27 (16.5)	
	Unknown	314 (69.2)	191 (65.9)	123 (74.5)	
**Surgery type**	Radical	87 (19.1)	49 (16.9)	38 (23.0)	.212
	Partial	193 (42.4)	129 (44.4)	64 (38.8)	
	No surgery	138 (30.3)	88 (30.3)	50 (30.3)	
	Other	37 (8.1)	24 (8.3)	13 (7.9)	
**Radiation**	Beam radiation	258 (56.7)	136 (46.9)	122 (73.9)	<.001
	Combination	3 (0.7)	1 (0.3)	2 (1.2)	
	None	175 (38.5)	136 (46.9)	39 (23.6)	
	Unknown	4 (0.9)	3 (1.0)	1 (0.6)	
	Recommended/unknown	11 (2.4)	11 (3.8)	0 (0.0)	
	Refused	4 (0.9)	3 (1.0)	1 (0.6)	
**Chemotherapy**	Yes	325 (71.4)	173 (59.7)	152 (92.1)	<.001
	No	130 (28.6)	117 (40.3)	13 (7.9)	
**Marital status**	Single	253 (55.6)	90 (31.0)	163 (98.8)	<.001
	Married	158 (34.7)	157 (54.1)	1 (0.6)	
	Divorced	11 (2.4)	11 (3.8)	0 (0.0)	
	Other	4 (0.9)	4 (1.4)	0 (0.0)	
**Median income**	Q1 (<$40,000–$54,999)	30 (6.6)	17 (5.9)	13 (7.9)	.979
	Q2 ($55,000–$74,999)	121 (26.6)	75 (25.9)	46 (27.9)	
	Q3 ($75,000–$94,999)	181 (39.8)	115 (39.7)	66 (40)	
	Q4 ($95,000–$120,000+)	123 (27.0)	83 (28.6)	40 (24.2)	

### Univariable analysis

Table 2, Table 3, Table 4 present univariable analysis of the risk factors for mortality for the entire cohort, adult patients, and pediatric patients, respectively. Race, sex, ethnicity, median household income, and time from diagnosis to treatment were not observed to have statistically significant effects on mortality on univariate analysis for any subgroup. In both age groups and the combined cohort, increasing age was an independent predictor of greater mortality risk (HR 1.03, p<.0001). For the combined cohort as well as the adult patient subset, a diagnosis of osteosarcoma (HR 2.19, p<.0001) and increased tumor size (HR 1.01, p=.002) were associated with increased risk of death, while receipt of radiation after surgery (HR 0.63, p=.00596) and partial tumor resection (HR 0.64, p=.005) were associated with decreased mortality risk. In the adult cohort specifically, radical tumor resection was associated with decreased risk of mortality (HR 0.5, p=.00527), while higher tumor grade (Grade III HR 2.73, p=.0314, Grade IV HR 2.39, p=.0486) was associated with increased risk of death. Interestingly, aside from patient age, none of the variables above were observed to have a statistically significant impact on mortality risk in the pediatric subgroup.

**Table 2:** Univariable and multivariable analysis of factors impacting survival for the whole cohort.

Parameter	Term	Univariate		Multivariate	
		HR (95% CI)	p value	HR (95% CI)	p value
**Age**	Age (continuous)	1.03 (1.02–1.03)	<.0001⁎	1.03 (1.01–1.04)	<.001⁎
**Sex**	Male vs. female (ref)	1.35 (1.02–1.78)	.037	1.49 (0.93–2.36)	.0952
**Race**	Black vs. White (ref)	1.17 (0.71–1.93)	.535	1.36 (0.56–3.3)	.495
	Asian/Pacific Islander/American Indian vs. White (ref)	1.23 (0.76–2)	.396	1.26 (0.55–2.92)	.586
**Hispanic ethnicity**	Hispanic vs. non-Hispanic	0.95 (0.69–1.33)	.781	1.01 (0.53–1.91)	.976
**Income quartile**	Q2: $55K–$74K vs. Q1: <$55K (ref)	0.89 (0.51–1.55)	.675	1.14 (0.45–2.86)	.781
	Q3: $75K–$94K vs. Q1: <$55K (ref)	1.1 (0.65–1.87)	.728	1.11 (0.46–2.69)	.813
	Q4: ≥$95K vs. Q1: <$55K (ref)	0.84 (0.48–1.48)	.549	0.7 (0.28–1.77)	.455
**Diagnosis**	Chondrosarcoma vs. Ewing’s sarcoma (ref)	1.6 (1.01–2.54)	.044⁎	0.7 (0.28–1.77)	.580
	Osteosarcoma vs. Ewing’s sarcoma (ref)	2.19 (1.65–2.92)	<.0001⁎	2.06 (1.17–3.61)	.0119⁎
**Grade**	Well differentiated; Grade I vs. moderately differentiated; Grade II (ref)	1.7 (0.69–4.18)	.246	0.61 (0.05–7.95)	.705
	Poorly differentiated; Grade III vs. moderately differentiated; Grade II (ref)	1.77 (0.76–4.13)	.189	1.72 (0.39–7.59)	.471
	Undifferentiated; anaplastic; Grade IV vs. moderately differentiated; Grade II (ref)	1.2 (0.53–2.73)	.664	2.59 (0.62–10.77)	.191
	Unknown vs. moderately differentiated; Grade II (ref)	1.25 (0.4–3.88)	.699	1.89 (0.47–7.53)	.368
**Tumor size**	Tumor size (mm)	1.01 (1–1.01)	.002⁎	1.01 (1–1.02)	.007⁎
**Surgery type**	Partial resection of tumor vs. no surgery (ref)	0.64 (0.47–0.88)	.005⁎	0.47 (0.19–1.17)	.104
	Radical resection of tumor vs. no surgery (ref)	0.68 (0.46–1)	.051	0.82 (0.29–2.3)	.709
	Surgery, not otherwise specified vs. no surgery (ref)	0.99 (0.48–2.06)	.985	0.85 (0.29–2.53)	.774
**Radiation**	Beam radiation vs. no radiation (ref)	0.68 (0.52–0.9)	.00596⁎	1.23 (0.49–3.09)	.661
**Radiation sequence**	Radiation after surgery vs. radiation before surgery/radiation with no surgery (ref)	0.63 (0.47–0.83)	.001⁎	0.76 (0.27–2.18)	.616
**Chemotherapy**	Chemotherapy vs. No chemotherapy (ref)	0.54 (0.41–0.72)	<.0001⁎	0.85 (0.43–1.69)	.646
**Time from diagnosis to treatment (d)**	Time from diagnosis to treatment (d)	1 (1–1.01)	.257	1 (0.99–1)	.256

HR, hazard ratio; CI, confidence interval.

⁎ Denotes statistical significance.

**Table 3:** Univariable and multivariable analysis of factors impacting survival for adult cases only.

Parameter	Reference	Univariate		Multivariate	
		HR (95% CI)	p value	HR (95% CI)	p value
**Age**	Age (continuous)	1.02 (1.01–1.03)	<.0001⁎	1.03 (1.01–1.04)	.00826⁎
**Sex**	Male vs. female (ref)	1.39 (1–1.94)	.0505	1.94 (1.07–3.52)	.0297⁎
**Race**	Black vs. White (ref)	1.07 (0.6–1.89)	.821	3.16 (1.07–9.34)	.0374⁎
	Asian/Pacific Islander/American Indian vs. White (ref)	1.16 (0.68–1.99)	.579	2.04 (0.7–5.91)	.191
**Hispanic ethnicity**	Hispanic	1.07 (0.7–1.64)	.764	1.18 (0.51–2.72)	.704
**Income quartile**	Q2: $55K–$74K vs. Q1: <$55K (ref)	1.14 (0.57–2.26)	.715	1.2 (0.39–3.67)	.747
	Q3: $75K–$94K vs. Q1: <$55K (ref)	1.14 (0.58–2.21)	.707	0.9 (0.31–2.64)	.854
	Q4: ≥$95K vs. Q1: <$55K (ref)	0.78 (0.39–1.57)	.489	0.4 (0.13–1.26)	.118
**Diagnosis**	Chondrosarcoma vs. Ewing’s sarcoma (ref)	1.08 (0.64–1.8)	.779	1.25 (0.51–3.05)	.629
	Osteosarcoma vs. Ewing’s sarcoma (ref)	1.67 (1.19–2.33)	.00276⁎	3.02 (1.55–5.88)	.0012⁎
**Grade**	Well differentiated; Grade I vs. moderately differentiated; Grade II (ref)	0.99 (0.28–3.51)	.985	1.49 (0.09–23.59)	.777
	Poorly differentiated; Grade III vs. moderately differentiated; Grade II (ref)	2.73 (1.09–6.79)	.0314⁎	2.01 (0.4–10.21)	.398
	Undifferentiated; anaplastic; Grade IV vs. moderately differentiated; Grade II (ref)	2.39 (1.01–5.68)	.0486⁎	4.87 (0.94–25.29)	.0597
	Unknown vs. moderately differentiated; Grade II (ref)	1.61 (0.7–3.7)	.265	3.26 (0.67–15.74)	.142
**Tumor size**	Tumor Size (mm)	1.01 (1–1.01)	.0111⁎	1.01 (1–1.02)	.0848
**Surgery type**	Partial resection of tumor vs. no surgery (ref)	0.51 (0.36–0.73)	.000191⁎	0.29 (0.09–0.92)	.0355⁎
	Radical resection of tumor vs. no surgery (ref)	0.5 (0.31–0.82)	.00527⁎	0.33 (0.1–1.07)	.0652
	Surgery, not otherwise specified vs. no surgery (ref)	0.83 (0.38–1.81)	.637	0.81 (0.21–3.12)	.756
**Radiation**	Beam radiation vs. no radiation (ref)	0.9 (0.65–1.24)	.507	1.16 (0.39–3.47)	.787
**Radiation sequence**	Radiation after surgery vs. radiation before surgery/radiation with no surgery (ref)	0.67 (0.48–0.95)	.0235⁎	0.82 (0.22–3.07)	.766
**Chemotherapy**	Chemotherapy vs. no chemotherapy (ref)	0.73 (0.54–1.01)	.054	0.69 (0.32–1.48)	.336
**Time from diagnosis to treatment**	Time from diagnosis to treatment (d)	1 (1–1)	.966	1 (0.99–1)	.212

HR, hazard ratio; CI, confidence interval.

⁎ Denotes statistical significance.

**Table 4:** Univariable and multivariable analysis of factors impacting survival for pediatric cases only.

Variable	Term	Univariate		Multivariate	
		HR (95% CI)	p value	HR (95% CI)	p value
**Age (y)**	Age (continuous)	1.1 (1.02–1.18)	.00938⁎	1.13 (1.03–1.23)	.00678⁎
**Sex**	Male vs. female (ref)	1.07 (0.63–1.82)	.816	1.18 (0.64–2.19)	.599
**Race**	Black vs. White (ref)	1.32 (0.47–3.66)	.6	0.84 (0.24–3.01)	.792
	Asian/Pacific Islander/American Indian vs. White (ref)	1 (0.31–3.22)	1.0	1.69 (0.36–8.02)	.509
**Hispanic ethnicity**	Hispanic	1.31 (0.75–2.28)	.347	1.29 (0.66–2.54)	.453
**Income quartile**	Q2: $55K–$74K vs. Q1: <$55K (ref)	0.37 (0.13–1.07)	.0678	0.76 (0.23–2.52)	.653
	Q3: $75K–$94K vs. Q1: <$55K (ref)	0.91 (0.38–2.22)	.839	1.3 (0.42–4.07)	.647
	Q4: ≥$95K vs. Q1: <$55K (ref)	0.83 (0.32–2.15)	.704	1.52 (0.45–5.12)	.503
**Diagnosis**	Chondrosarcoma vs. Ewing’s sarcoma (ref)⁎⁎***N*=5**	3.18 (0.98–10.33)	.055	15.41 (2.62–90.63)	.00248⁎
	Osteosarcoma vs. Ewing’s sarcoma (ref)	1.59 (0.78–3.27)	.204	1.18 (0.4–3.48)	.77
**Grade**	Undifferentiated; anaplastic; Grade IV vs. poorly differentiated; Grade III (ref)	2.09 (0.58–7.5)	.258	0.82 (0.19–3.46)	.787
	Unknown/Other vs. poorly differentiated; Grade III (ref)	1.8 (0.56–5.83)	.325	1.06 (0.29–3.89)	.93
**Surgery type**	Partial resection of tumor vs. no surgery (ref)	0.86 (0.44–1.71)	.669	1.07 (0.27–4.31)	.925
	Radical resection of tumor vs. no surgery (ref)	1.37 (0.69–2.74)	.373	1.75 (0.45–6.79)	.417
	Surgery, not otherwise specified vs. no surgery (ref)	1.9 (0.74–4.89)	.181	1.98 (0.44–8.79)	.371
**Radiation**	Beam radiation vs. no radiation (ref)	0.71 (0.39–1.29)	.261	0.71 (0.18–2.74)	.615
**Radiation sequence**	Radiation after surgery vs. radiation before surgery/radiation with no surgery (ref)	0.83 (0.49–1.41)	.494	0.81 (0.19–3.44)	.771
**Chemotherapy**	Chemotherapy vs. no chemotherapy (ref)	0.53 (0.22–1.23)	.137	1.08 (0.21–5.47)	.924
**Time from diagnosis to treatment (d)**	Time from diagnosis to treatment (d)	1 (0.97–1.03)	.921	0.98 (0.95–1.02)	.31

HR, hazard ratio; CI, confidence interval.

⁎ Denotes statistical significance.

⁎⁎ Denotes poor statistical power

### Multivariable analysis

Table 2, Table 3, Table 4 also present multivariable CPHM of risk factors for mortality for the entire cohort, adult patients, and pediatric patients, respectively. Ethnicity, median household income, receipt of radiation, radiation/surgery sequence, tumor grade, and time from diagnosis to treatment were not observed to have statistically significant effects on mortality on multivariable analysis for any subgroup. Age proved a durable predictor of mortality; again, increased age was associated with an increased risk of death across all analyses (HR 1.03, p<.0001). For adult patients as well as the entire cohort, a diagnosis of osteosarcoma was associated with increased risk of mortality (entire cohort: HR 2.06, p=.0119, adults: HR 3.02, p=.0012). For adults only, Black race and male sex decreased likelihood of survival (HR 3.16, p=.0374; HR 1.94, p=.0297) while surgical resection status was significantly associated with survival, with patients undergoing partial resection demonstrating better outcomes than those receiving no surgery (HR 0.29, p=.0355). In the pediatric subgroup, no variable aside from age showed any significant association with survival likelihood aside from a diagnosis of chondrosarcoma, which was associated with increased mortality risk; however, the utility of this finding is limited by a small sample size (*N*=5).

### Kaplan–Meier survival analysis

**Fig. 1:**
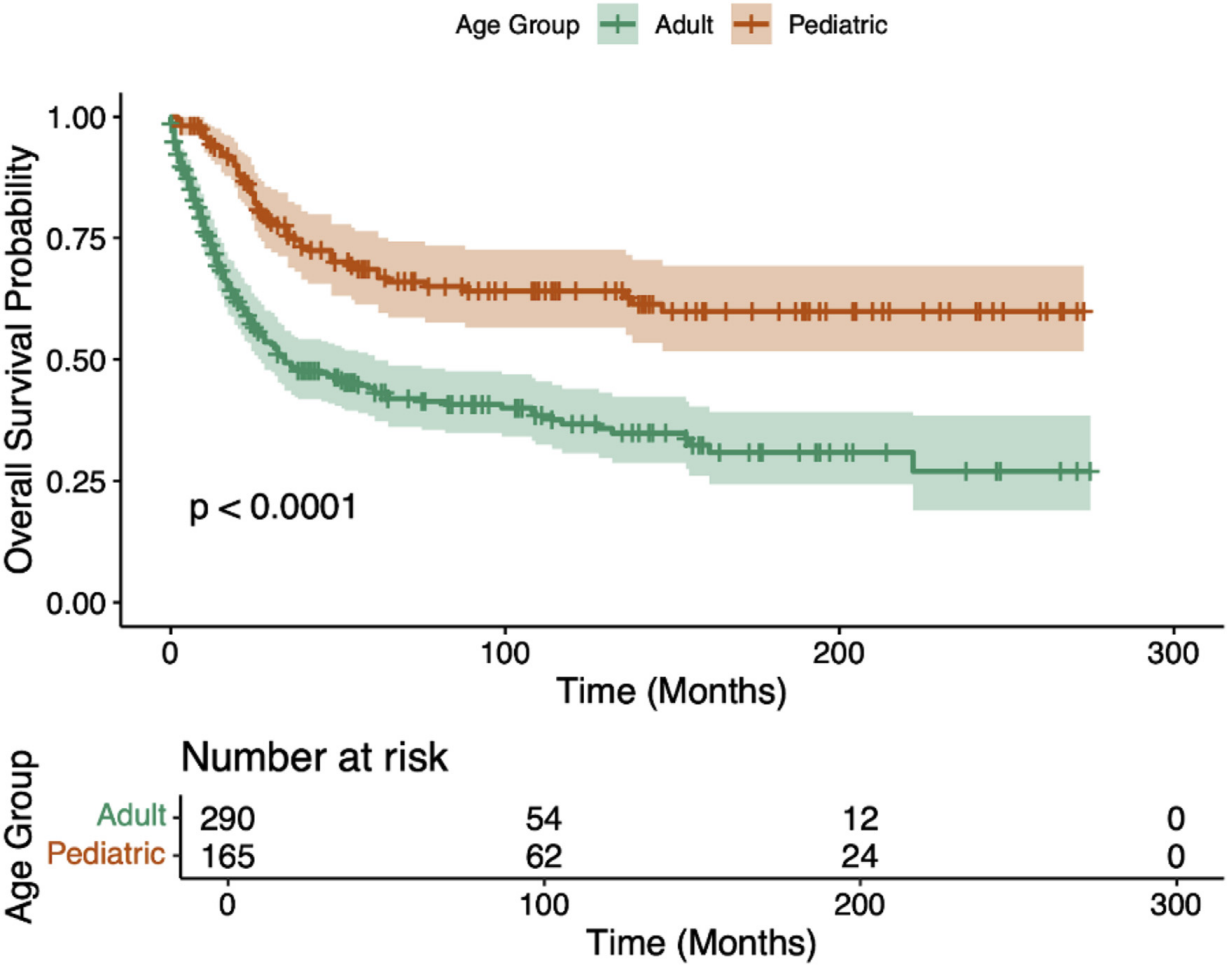
Kaplan–Meier plot of cancer-specific survival stratified by age group.

**Fig. 2:**
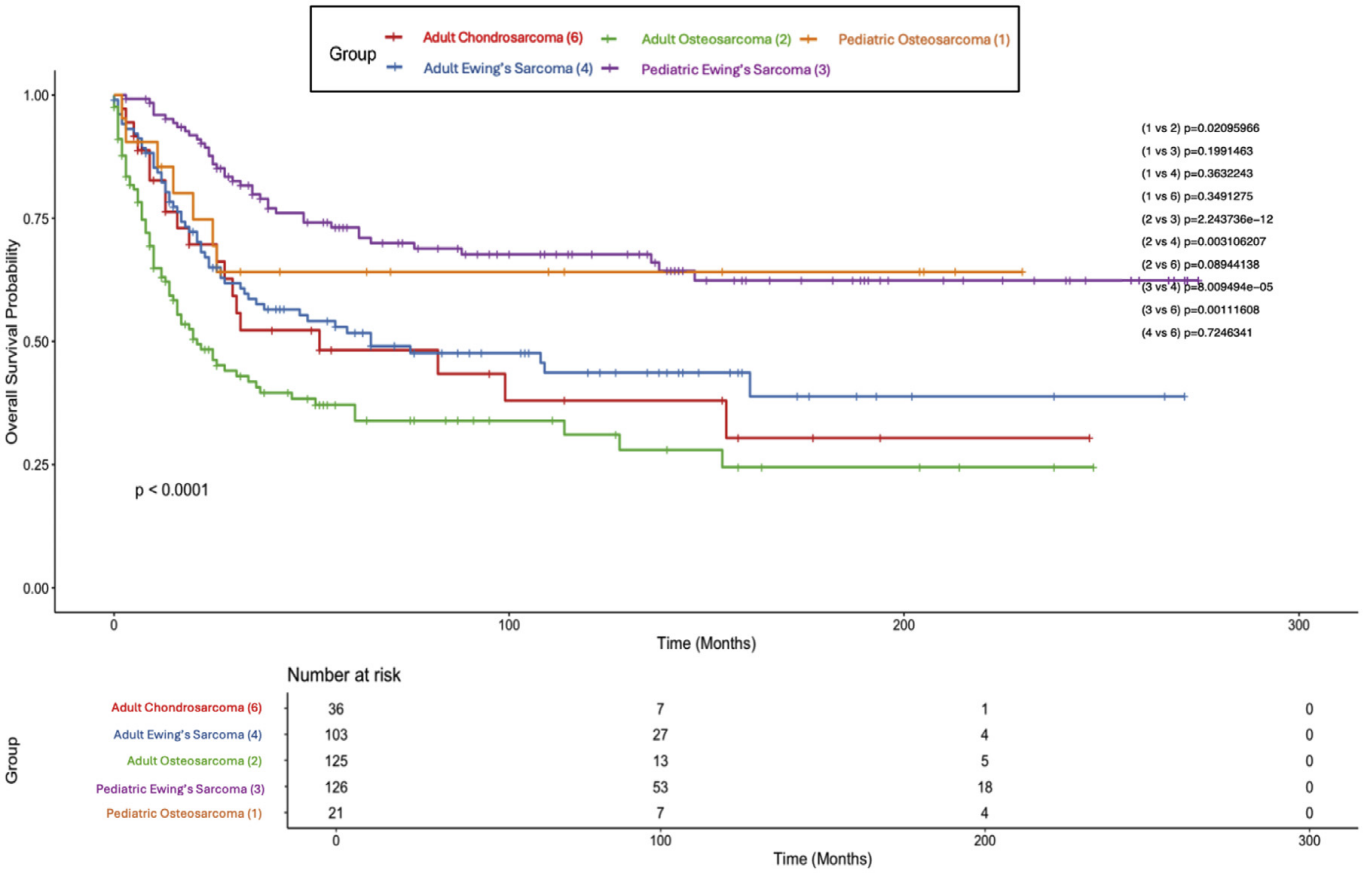
Kaplan–Meier plot of cancer-specific survival stratified by age group and diagnosis.

**Fig. 3:**
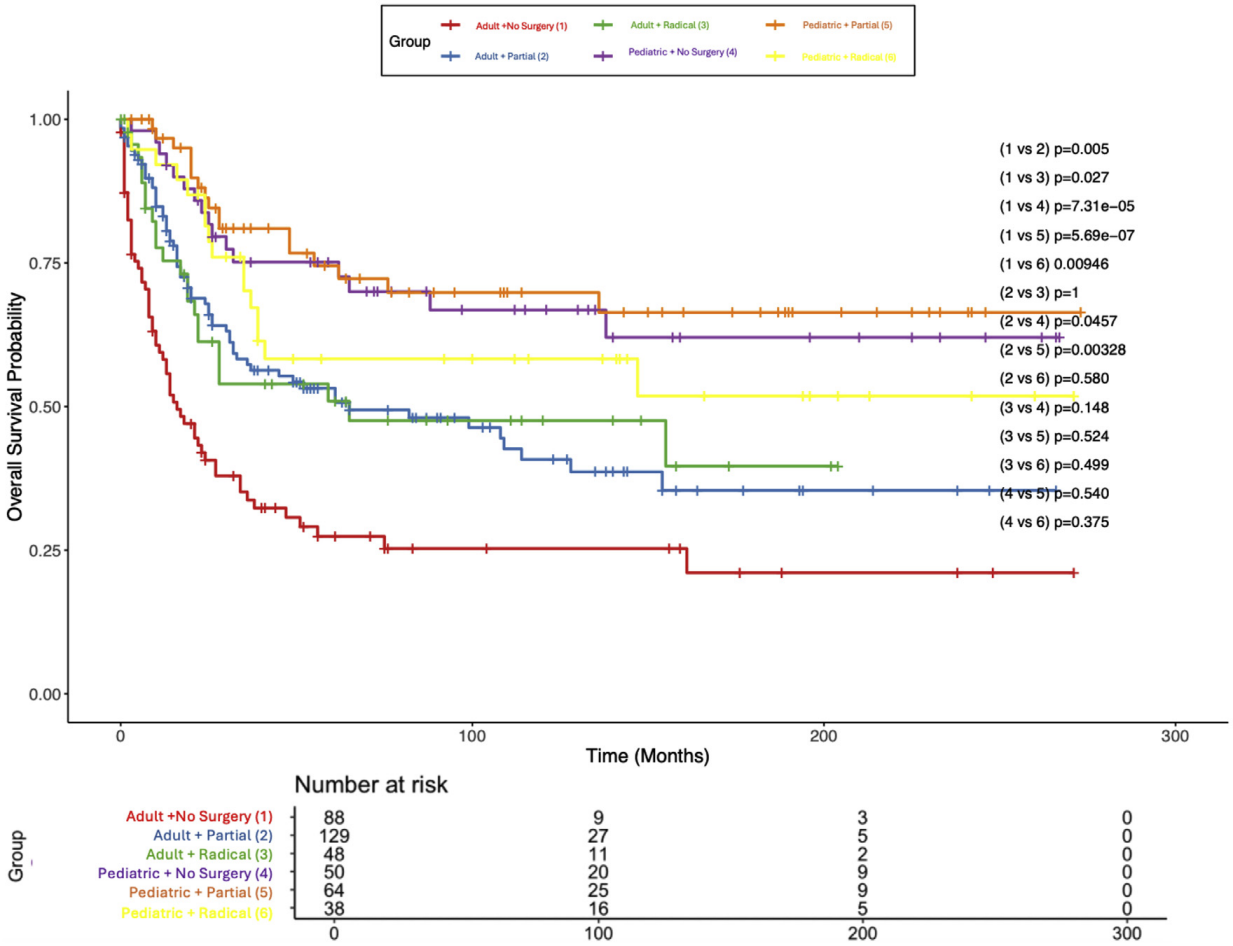
Kaplan–Meier plot of cancer-specific survival stratified by age group and surgery type.

In the entire cohort (*N*=455), median CSS was 82 months (95% CI 52–154 months) and median OS was 62 months (95% CI 47–112 months). For the whole cohort, the 5-year CSS was 53% (5-year OS = 51.3%) and a 10-year CSS of 47% (10-year OS = 43.6%). For adults, median CSS was 34 months (95% CI 26–61 months) and median OS was 32 months (95% CI 24–52 months); the 5-year CSS was 44% (5-year OS = 42.4%), and the 10-year CSS was 37% (10-year OS = 34.3%). The median CSS or OS was not reached in the pediatric cohort, with a 69% 5-year CSS (66.1% 5-year OS) and a 64% 10-year CSS (58.9% 10-year OS). CSS differences between adults and children are compared in Fig. 1, with adults having a worse prognosis (p<.0001). Fig. 2 displays CSS of children and adults stratified by diagnosis; children with Ewing’s sarcoma fared the best, with adults diagnosed with osteosarcoma experiencing the worst outcomes. Survivorship was similar across pediatric patients and was superior to adults irrespective of diagnosis; however, adults with Ewing’s sarcoma fared better than adults with osteosarcoma (p=.0311). Fig. 3 displays CSS of children and adults stratified by surgery type. Again, children fared far better than their adult counterparts, and no surgery type (radical, partial, or no surgery) was associated with any statistically significant survival advantage. However, partial or radical resection was associated with significantly better survival in adults compared to no surgery (p=.005 and p=.027, respectively).

## Discussion

### Summary of findings

Our study identified several prognostic factors that varied between pediatric and adult patients with primary osseous sarcomas of the spine. Increasing age and osteosarcoma histology were independently associated with greater mortality risk in both univariable and multivariable analysis for the entire cohort and adults. Partial resection was linked to a survival advantage in adults after controlling for covariates (p=.00248). Aside from increased hazard of Black race (p=.0374) and male sex (p=.0297) in adults, patient sex, race, ethnicity, income, and time to treatment were not associated with mortality in any other statistical analysis.

Kaplan–Meier analysis revealed a median CSS of 82 months across the entire cohort, with 5- and 10-year survival rates of 53% and 47%, respectively. Adult patients had a median CSS of 34 months, with 5- and 10-year survival rates of 44% and 37%, while the pediatric cohort did not reach median survival, with 5- and 10-year CSS rates of 69% and 64%. Stratification by histologic subtype showed that pediatric patients with Ewing’s sarcoma had the most favorable outcomes, while adults with osteosarcoma experienced the poorest survival. Adults with Ewing’s sarcoma fared significantly better than those with osteosarcoma (p=.005). With regards to surgery type, partial and radical resection of tumors was associated with significantly greater survival compared to no surgery in the adult population (p=.005 and p=.027, respectively). In the pediatric subgroup, aside from age, no variables demonstrated a statistically significant association with mortality, although chondrosarcoma was linked to worse outcomes in a small sample.

These results likely are observed for a few distinct and relevant reasons. The data suggesting the finding that pediatric patients fare better than adult patients is expected; prior research has shown that patient age is an independent prognostic indicator in both osseous and soft tissue sarcoma cohorts [5,17]. This could be due to an increased capability of pediatric patients to tolerate intensive chemotherapy regimens, or an inherent biologic difference in tumor characteristics, rather than a treatment-related effect. Furthermore, patients eligible for surgical resection may have lower-grade tumors without significant vascular or neurologic infiltration at presentation, while higher-grade tumors with metastatic disease would make safe surgical intervention challenging or impossible. Therefore, patients who received surgery may have had a less severe disease course at outset. The data suggesting that Black race is associated with increased mortality risk in adults could be representative of wider structural inequities in the United States healthcare system, such as access to care or treatment delays, as well as increased comorbidity burden [18].

### Comparison with prior literature

While prior studies on primary osseous sarcomas have often focused on nonspinal cases or included a mixture of spinal and extraspinal tumors, our analysis offers spine-specific insight into the prognostic implications of histological subtype and age. In doing so, our findings both align with and diverge from the existing literature in several meaningful ways.

Consistent with prior retrospective studies, we found that Ewing sarcoma was associated with the most favorable survival, particularly in pediatric patients [9,19]. Arshi et al. [20] echoed this trend in a larger SEER cohort, demonstrating superior 5-year OS for Ewing sarcoma relative to osteosarcoma (44% vs. 18%) and a positive impact of surgical resection in both subtypes. In contrast, osteosarcoma consistently conferred the poorest prognosis among the major histologic subtypes in our cohort. This agrees with existing literature on axial osteosarcoma, which has recognized the malignancy’s aggressive nature. Wang et al. [21], for example, identified both osteosarcoma histology and tumor size as strong independent predictors of poor cancer-specific survival in children. Although our analysis of spinal chondrosarcoma was limited by a smaller sample size, it nonetheless offers valuable context. The greater prevalence of chondrosarcoma among adults vs. pediatric patients (13.1% vs. 3.0%, p<.001) is consistent with prior reports indicating that spinal chondrosarcoma is rare in children [22]. Within the pediatric subgroup, chondrosarcoma was associated with markedly worse survival relative to Ewing sarcoma (HR 15.41, 95% CI: 2.62–90.63, p=.0025), though this result should be interpreted cautiously given the small sample size (*n*=5). In the overall cohort, univariate analysis revealed a higher mortality hazard for chondrosarcoma compared to Ewing sarcoma (HR 1.60, p=.044), though this association diminished after multivariable adjustment, likely reflecting confounding by age or treatment.

These trends are broadly consistent with analyses such as those by Boriani et al., who reported improved local control and OS with en bloc resection in spinal chondrosarcoma, and Hsieh et al., who found disease-free survival extended to 51 months following wide excision vs. only 17.5 months with intralesional resection [23,24]. Importantly, our SEER-based dataset highlights a major limitation in achieving ideal oncologic management: gross total resection was achieved in just 19.1% of cases, and nearly 30% of patients did not undergo surgery at all, reflecting the substantial anatomical and morbidity-related barriers inherent to aggressive resection of spinal tumors. In support of the literature, in the adult population, we found that both GTR and STR were associated with improved CSS. However, our analysis did not yield any statistically significant association between resection extent and CSS in the pediatric subgroup. This discrepancy between our findings and prior literature may be due to a smaller number of pediatric cases in our study and limited statistical power. In practice, the high surgical morbidity risk in children may limit aggressive resection, but further research is necessary to further clarify the role of surgical intervention in children with spinal osseous sarcomas with larger cohorts.

Beyond the role of resection extent, few prior studies have directly compared the survival outcomes of primary osseous sarcomas of the spine between pediatric and adult patients. Our analysis found that pediatric patients demonstrated significantly improved CSS regardless of surgical intervention or histological subtype. Studies of both spinal and extraspinal osteosarcoma and Ewing’s sarcoma similarly identified younger age as an independent protective factor when adjusting for tumor size, stage, primary site, and histological subtype [20,21]. Despite the novel insights provided by our analysis, further investigation is warranted given the limitations inherent to using a SEER-based database. Overall, our findings provide spine-specific insight into the prognostic impact of age, histology, and surgical intervention in primary osseous sarcomas, generally aligning with established trends while also suggesting distinctions that merit further investigation in larger, more granular studies.

### Clinical implications

The present population-level analysis yields several clinical implications. Expanding upon prior general studies of sarcomas, we found that pediatric patients with primary osseous sarcomas of the spine experience significantly improved OS compared to adults, independent of histological subtype. In a clinical context, our results support the consideration of both patient age and tumor biology when determining prognosis, particularly in spine-specific cases, and they corroborate prior findings from more general, extraspinal tumor studies.

In adult patients, we found that osteosarcoma histology, advanced tumor grade, and the absence of surgical resection were associated with worse OS. Conversely, we observed that both partial and radical resection conferred a survival benefit. These findings provide evidence in support of a more aggressive surgical approach in adult patients presenting with primary osseous sarcomas of the spine, when clinically appropriate.

In contrast, survival outcomes in pediatric patients were not significantly influenced by extent of resection or adjuvant treatment variables in our multivariable analysis. While this may in part reflect limitations in sample size and statistical power, our findings suggest that pediatric spinal sarcomas may exhibit distinct treatment responses. As such, treatment paradigms for pediatric cases should emphasize individualized decision-making that carefully balances aggressive tumor control with the potential for postoperative morbidity.

Overall, our study suggests that age and histology are key factors to consider when optimizing outcomes in osseous sarcomas of the spine. Given that nearly one-third of adult patients in our study did not undergo surgery, our findings also highlight an opportunity to investigate potential disparities in access to timely surgical care, particularly given the observed survival benefit. Multidisciplinary management that integrates histologic subtype, patient demographics, and appropriate surgical planning may help improve outcomes in this rare but aggressive disease.

### Future directions and limitations

The present analysis has several key limitations. The SEER database offers population-level data with survival as the primary outcome variable, but lacks other key treatment data and outcomes. Given that such factors may both notably influence survival outcomes and are useful clinical variables in their own right, our SEER-based study is limited in its applicability. Another significant limitation is that a large proportion of patients within this subset of the SEER database (>66%) had unknown tumor grade, putting a constraint on this study’s ability to speak to grade as a prognostic indicator. This issue may also confound the perceived association between tumor resection and survival benefit; lower-grade, well-circumscribed tumors are likely more amenable to resection, while patients with higher-grade tumors may not be surgical candidates. Thus, future studies should strive to incorporate tumor grade into initial data collection so that more granular subgroup analyses may be conducted. Additionally, other detailed clinical variables such as tumor stage, medical comorbidities, performance status, neurologic status at presentation, chemotherapy dose, radiation type, and surgical approach are not available, increasing the risk of confounding variables in survival analyses. Histological classification in SEER is dependent on registry coding, rather than pathologist review. The rarity of primary osseous sarcomas of the spine also contributed to a modest sample size in our study, with pediatric subgroups having a particularly restricted sample size. Such limitations prevented our ability to maintain stability in multivariable Cox regression for some covariates, and our findings may have been underpowered to detect statistically significant findings. Future studies should focus on prospective multicenter registries that include detailed clinical, treatment-related, and tumor genomic data. Such analysis would offer more advanced risk stratification and may help better explain any age-related differences in survival outcomes for primary osseous sarcoma. Beyond survival, further work should also investigate patient-reported outcome data and functional recovery, which are important factors to consider when treating these aggressive malignancies.

## Conclusion

In this population-based analysis of primary osseous sarcomas of the spine, we found that pediatric patients experienced significantly improved CSS compared to adults, independent of histological subtype or treatment modality. In adult patients, osteosarcoma histology, higher tumor grade, male sex, black race, and absence of surgical resection were associated with worse outcomes, while partial and radical resections were linked to a survival benefit. In contrast, survival in pediatric patients was not significantly influenced by treatment-related variables in multivariable analysis, suggesting potential differences in tumor biology or treatment response. Overall, our findings suggest the importance of considering both patient age and histology when determining prognosis and treatment strategy. Further research using more granular clinical and molecular data is warranted to refine risk stratification and guide optimal care for these rare spinal malignancies.

## Ethical statement

This study is considered IRB exempt.

## Data availability statement

Data used in this study are publicly available via the SEER cancer registry.

## Funding

We have received no funding for this research.

## Declarations of competing interests

The authors declare that they have no known competing financial interests or personal relationships that could have appeared to influence the work reported in this article.
